# Islet Oxygen Consumption Rate (OCR) Dose Predicts Insulin Independence in Clinical Islet Autotransplantation

**DOI:** 10.1371/journal.pone.0134428

**Published:** 2015-08-10

**Authors:** Klearchos K. Papas, Melena D. Bellin, David E. R. Sutherland, Thomas M. Suszynski, Jennifer P. Kitzmann, Efstathios S. Avgoustiniatos, Angelika C. Gruessner, Kathryn R. Mueller, Gregory J. Beilman, Appakalai N. Balamurugan, Gopalakrishnan Loganathan, Clark K. Colton, Maria Koulmanda, Gordon C. Weir, Josh J. Wilhelm, Dajun Qian, Joyce C. Niland, Bernhard J. Hering

**Affiliations:** 1 Institute for Cellular Transplantation, Department of Surgery, University of Arizona, Tucson, Arizona, United States of America; 2 Department of Surgery, University of Minnesota, Minneapolis, Minnesota, United States of America; 3 Schulze Diabetes Institute, Minneapolis, Minnesota, United States of America; 4 Department of Chemical Engineering, Massachusetts Institute of Technology, Cambridge, Massachusetts, United States of America; 5 The Transplant Institute, Beth Israel Deaconess Medical Center (BIDMC), Harvard Medical School, Boston, Massachusetts, United States of America; 6 Joslin Diabetes Center, Boston, Massachusetts, United States of America; 7 Information Science, City of Hope, Duarte, California, United States of America; La Jolla Institute for Allergy and Immunology, UNITED STATES

## Abstract

**Background:**

Reliable *in vitro* islet quality assessment assays that can be performed routinely, prospectively, and are able to predict clinical transplant outcomes are needed. In this paper we present data on the utility of an assay based on cellular oxygen consumption rate (OCR) in predicting clinical islet autotransplant (IAT) insulin independence (II). IAT is an attractive model for evaluating characterization assays regarding their utility in predicting II due to an absence of confounding factors such as immune rejection and immunosuppressant toxicity.

**Methods:**

Membrane integrity staining (FDA/PI), OCR normalized to DNA (OCR/DNA), islet equivalent (IE) and OCR (viable IE) normalized to recipient body weight (*IE dose* and *OCR dose*), and OCR/DNA normalized to islet size index (ISI) were used to characterize autoislet preparations (n = 35). Correlation between pre-IAT islet product characteristics and II was determined using receiver operating characteristic analysis.

**Results:**

Preparations that resulted in II had significantly higher *OCR dose* and *IE dose* (p<0.001). These islet characterization methods were highly correlated with II at 6–12 months post-IAT (area-under-the-curve (AUC) = 0.94 for *IE dose* and 0.96 for *OCR dose*). FDA/PI (AUC = 0.49) and OCR/DNA (AUC = 0.58) did not correlate with II. OCR/DNA/ISI may have some utility in predicting outcome (AUC = 0.72).

**Conclusions:**

Commonly used assays to determine whether a clinical islet preparation is of high quality prior to transplantation are greatly lacking in sensitivity and specificity. While *IE dose* is highly predictive, it does not take into account islet cell quality. *OCR dose*, which takes into consideration both islet cell quality and quantity, may enable a more accurate and prospective evaluation of clinical islet preparations.

## Introduction

Cell-based transplant therapies may offer a minimally-invasive alternative to solid organ transplants for certain diseases such as diabetes. Islet transplantation is emerging as an attractive therapy [1–4]. There is a critical need for reliable assays that assess the quality of clinical islet preparations prior to transplantation, especially as islet transplant is more widely-applied and becoming standard-of-care. Such assays would prevent transplantation of islet preparations of poor quality that would fail to reverse diabetes and would facilitate regulatory approvals. Currently-accepted product release criteria for clinical islet allotransplant include the islet dose based on number of islet equivalents (IE) normalized to the recipient body weight (IE/kg), fractional viability as estimated with membrane integrity staining using fluorescein diacetate/propidium iodide (FDA/PI), islet purity based on dithizone (DTZ) staining, and glucose responsiveness as measured by static insulin release assays [5, 6]. However, clinical transplant outcomes (CTOs) cannot be discerned based on these pre-transplant criteria [6]. These currently-available quality assessment assays are not fully reliable, exhibiting high false positive rates when attempting to predict rates of insulin independence (II).

**Fig 1:**
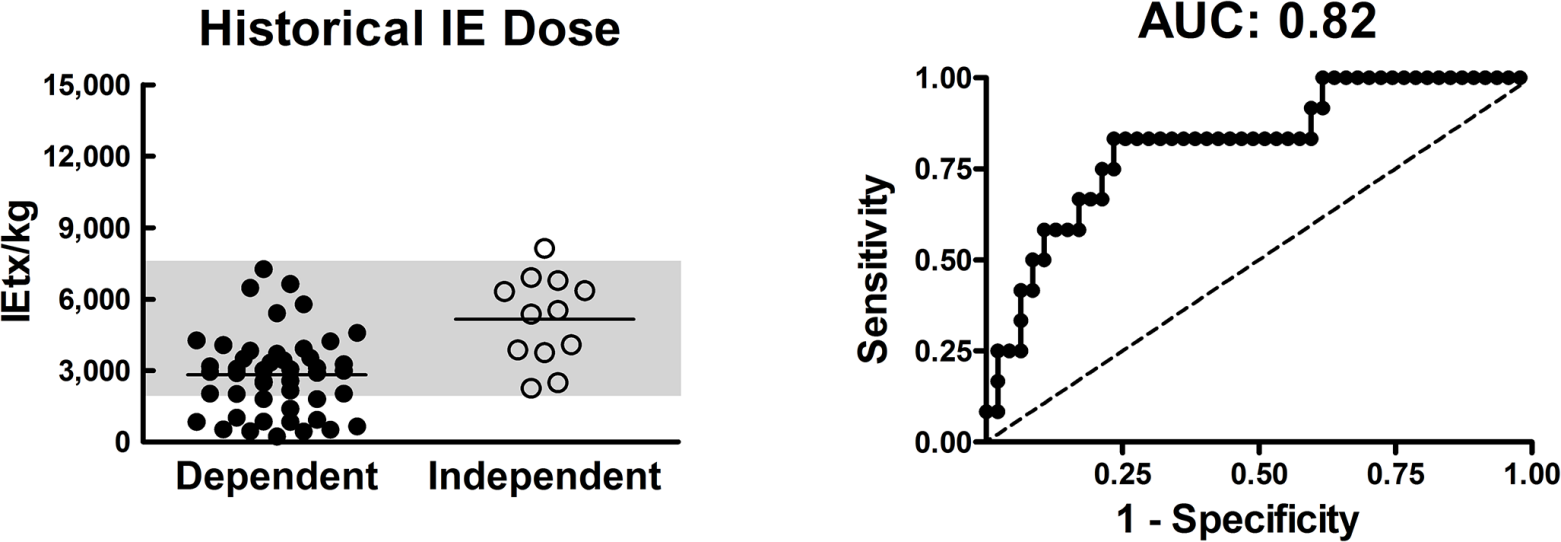
Previously published data showing overlap and correlation of islet equivalent dose with clinical transplant outcome. Data from Anazawa *et al*. [10] illustrating that the *islet equivalent* (*IE*) *dose* correlates with the clinical outcome [insulin independence vs. dependence] at 6–12 months following islet autotransplant (IAT). However, the gray region indicates a wide range of *IE dose* (IE/kg of recipient) that is associated with an uncertain IAT outcome. The second graph shows the receiver operating characteristic (ROC) curve for this previously published data set with the area-under-the-curve (AUC) above.

Islet dose normalized to recipient body weight presented in units of IE/kg has been reported to correlate with clinical islet auto- and allotransplant outcomes [7–9] and threshold doses have been established to try to increase the likelihood of a favorable CTO. However, there have been numerous cases in which very high *IE doses* have failed to reverse diabetes, while low doses were successful. Previously published intraportal islet autotransplant (IAT) data from the University of Minnesota (n = 59) have shown that the *IE dose* correlates with CTOs [Fig 1, receiver operating characteristic (ROC) analysis yields area-under-the-curve (AUC) of 0.82] [10]. However, there was a high degree of overlap in *IE doses* resulting in either II or dependence, as shown by the grey bar region in Fig 1. We hypothesized that the viable *IE dose* (or *OCR dose*) may be more predictive of CTOs than the total *IE dose*. We utilized oxygen consumption rate (OCR) measurements normalized to DNA (OCR/DNA, a measure of fractional viability) to estimate the *OCR dose* (the product of the *IE dose* and OCR/DNA). To test this hypothesis, we utilized the clinical IAT model, which is an attractive model for evaluating characterization assays due to an absence of confounding factors such as immune rejection or immunosuppressant toxicity.

OCR has been proposed as a means to evaluate islet quality [6, 11–15]. OCR measurements performed using the stirred microchamber system are accurate, quantitative, operator-independent, and can be performed prospectively [13–15]. Since the technology has been developed, data have been generated that correlate islet OCR and OCR/DNA measurements (when these parameters are used in combination) and transplant outcomes with the nude mouse bioassay [13, 15]. In these animal studies, graft function (or non-function) could be associated with discrete regions defined by measurements of OCR and OCR/DNA done on islet preparations prior to transplant. Recently published work by our group showed that *OCR dose* was able to predict transplant outcome in a small number of cases using the human allotransplant model (donor islets transplanted into type 1 diabetic recipients) [16]. The present study is the first publication to explore the use of *OCR dose* to predict CTO following islet autotransplantation (non-diabetic pancreatitis patients transplanted with their own islets).

## Materials and Methods

### Study Design

Data from patients experiencing severe pancreatitis who underwent total pancreatectomy (TP) and IAT at the University of Minnesota from June 2005 to October 2009 were studied herein. Only patients with 6–12 months of post-IAT follow-up and available pre-IAT OCR measurements were included in the analysis (n = 35). Exclusion criteria were pre-existing diabetes mellitus or impaired glucose tolerance, or prior partial pancreatectomy with IAT. II was defined as fasting blood glucose level (BGL) <126 mg/dL, 2-hour postprandial BGL <180 mg/dL, and hemoglobin A1C (HbA1C) ≤6.5% without use of exogenous insulin. We examined the utility of 5 different pre-IAT islet product characteristics [FDA/PI, OCR/DNA, OCR/DNA normalized to the islet size index (ISI), *IE dose* and *OCR dose*] to predict post-IAT II rates.

### Ethics statement

The present study was approved by the University of Minnesota Institutional Review Board (UMN-IRB). Patients transplanted prior to September 2006 were covered under UMN-IRB protocol #0511M77516 with verbal consent obtained by telephone. The patient was mailed the informed consent in advance, and the investigator obtained informed consent subsequently by telephone. This study was a retrospective assessment of outcomes which included medical records review and a telephone questionnaire. Because patients did not have any onsite study visit, and the study risk was minimal, the UMN-IRB approved obtaining consent by telephone as described. Patients transplanted following September 2006 were covered under UMN-IRB protocol #0609M91887 with written consent obtained and documented on an informed consent form and in the patient’s medical records.

### Total pancreatectomy, islet isolation and purification

TP-IAT was performed as described previously [17]. Briefly, TP was done to preserve the blood supply to the pancreas until immediately before resection to minimize warm ischemia time. Following TP, the pancreas was surface-cooled and transported to the islet isolation laboratory. The basic method for islet isolation and purification has been described previously [17]. Each pancreas was distended by an intraductal infusion of collagenase solution and then digested at 37°C using the Ricordi method [18]. After digestion, the islet preparation may have been additionally purified by density gradient centrifugation. The decision to purify the islet preparation was based on the post-digest tissue volume (TV), with >0.25 cm^3^/kg (volume per kilogram recipient body weight) generally serving as an indication for purification.

### Islet product characterization

Aliquots of islet product were manually counted using an inverted light microscope to estimate the total number of islet particles (IPs) of >50 μm in diameter in the entire preparation. Based on stratifying each IP into an approximate size range (50–100 μm, 100–150 μm, etc.) during the manual count, the total number of IPs in the preparation was converted to a total number of IEs to account for differences in islet size. This was done by assuming that a perfectly spherical 150 μm diameter islet represented the volume of 1 IE [5]. The total number of IEs per kilogram recipient body weight (IE/kg) was defined as the *IE dose*. A visual estimate of purity was performed by estimating the percent of insulin-producing cells stained by DTZ [19]. Furthermore, ISI (a dimensionless quantity) was calculated by dividing the total number of IEs in a preparation by the total number of IPs. ISI provides a simple metric that reflects the average size of an IP in a preparation [20, 21].

All preparations were evaluated for their fractional viability by differential membrane integrity staining with FDA/PI [5]. OCR/DNA was measured approximately 1–4 hours post-isolation when sufficient IE were isolated; the technique has been previously described in detail [13–15]. Average fractional viabilities from multiple measurements were reported (n = 2 to 4). OCR/DNA is a measure of fractional viability since DNA is stable, even in dying tissue over >24 hours [22], and its content per IE is reliably constant [5]. Based on promising data in a recent literature report, the mean OCR/DNA value of an islet preparation was normalized to the ISI, giving a separate pre-IAT quality assessment metric of OCR/DNA/ISI (with same units as OCR/DNA; $\frac{nmol\;O_{2}}{min∙mg\;DNA}$) [23].

The *viable IE dose* (or *OCR dose*) was calculated from the *IE dose* and OCR/DNA. *IE dose* was converted to an amount of DNA after multiplying by the conversion factor of 10.4 ng DNA per IE [5]. The DNA dose in the transplanted preparation was then multiplied by the mean OCR/DNA value to yield an *OCR dose* (in units of nmol O_2_/min•kg recipient body weight; $\frac{nmol\;O_{2}}{min∙mg\;DNA}\times \frac{10.4\;ng\;DNA}{IE}\times \frac{mg}{1x10^{6}\;ng}\times \frac{IE}{kg}$).

### Islet autotransplantation

The typical autoislet product preparation time ranged from 3.5–6.5 hours. Islet products were suspended in Connaught Medical Research Laboratories (CMRL)-1066 transplant medium (Mediatech Inc., Manassas, VA) supplemented with 25mM HEPES, antibiotics, and 2.5% m/v human serum albumin and transferred to the operating room. Heparin (70 units/kg) was administered systemically and the islet product was directly infused under gravity into the portal vein over a 15–60 minute period. During the infusion, the portal pressures were continuously monitored and if they exceeded 25 cm H_2_O the remainder of the islet preparation was infused into the peritoneal cavity. All patients were placed on an insulin drip in the immediate post-operative period, which was transitioned to subcutaneous insulin injections at ~1 week post-operatively. Target BGLs have been summarized elsewhere [24].

### Statistical analysis

Relationships between pre-IAT islet product characteristics and CTOs were examined using ROC curve analysis. The AUC was calculated from ROC curves generated for each islet product characteristic and these values were evaluated. We analyzed several multivariate models and additionally included age, BMI, and purity to the model. A backward selection was used and we assessed the goodness of the models with the AUC of the ROC curve. In addition, we compared the models using the Akaike information criterion (AIC) values with a smaller AIC value standing for a better model. Statistical significance corresponded to *p*-values <0.05. All statistical analyses were performed using SAS statistical software package, version 9.3 (SAS Institute Inc., Cary, NC) or GraphPad Prism Version 5.03 (GraphPad Software Inc., La Jolla, CA).

## Results

**Fig 2:**
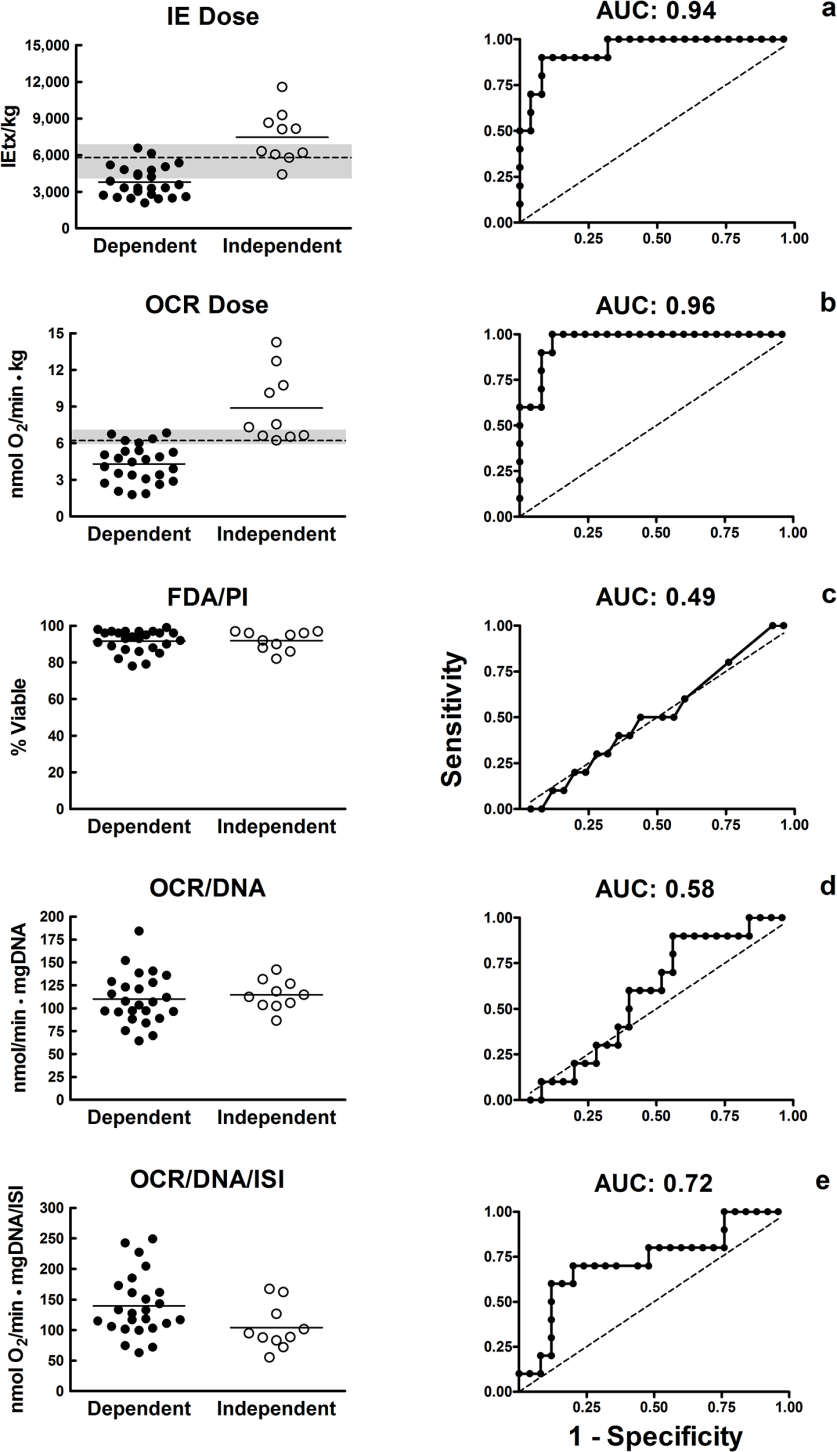
Overlap and correlation of islet characterization methods with clinical transplant outcome. Data from our study illustrating that membrane integrity staining (based on FDA/PI), oxygen consumption rate (OCR) normalized to DNA content (OCR/DNA), and OCR/DNA normalized to the islet size index (ISI) (OCR/DNA/ISI) are not correlated with the clinical outcome [insulin independence vs. dependence] at 6–12 months following islet autotransplant (IAT). However, both *islet equivalent* (IE) *dose* and the *OCR dose* were correlated with post-IAT outcome. The gray region indicates the range of *IE* and *OCR doses* that is associated with uncertain IAT outcome. Note that the width of the gray region is much narrower with the *OCR dose*. The black dotted line represents the calculated cut-off point for clinical outcome (*IE dose*: 5,794 and *OCR dose*: 6.23). The second column of graphs represents receiver operating characteristic (ROC) curves for each of the five islet product characteristics from this data set. The area-under-the-curve (AUC) has been calculated for each islet product characteristic and these values are shown above each ROC curve.

There were significant differences in BMI (*p* = 0.006), IE/g trimmed pancreas weight (p = 0.05), purity (*p* = 0.02), and ISI (*p =* 0.009) between patients achieving II versus those with insulin dependence 6–12 months post-IAT (Table 1). For islet characterization methods, there were no significant differences in FDA/PI (*p* = 0.96) or OCR/DNA (*p* = 0.48). However, there were significant differences in the OCR/DNA/ISI (*p* = 0.043), *IE dose* (*p*< 0.001) and *OCR dose* (*p*< 0.001) between groups. The grey regions in Fig 2 illustrate values of overlap between CTO that may poorly predict post-IAT outcomes for IE/kg and OCR/kg. More cases fell in the grey region of uncertainty for IE/kg (n = 13) than OCR/kg (n = 7). In cases #25, 32, 33 and 35 which had a high *IE dose* that did not result in II, the *OCR dose* was able to correctly classify the CTO (Table 2). The same was true in case #11 with low *IE dose* and high *OCR dose* in a patient achieving II. For *IE dose*, the rate of correct estimation was 94% with a sensitivity of 0.9 and specificity of 0.92, and the calculated cut-off point for CTO was 5,794 IE/kg. For *OCR dose*, the rate of correct estimation was 96% with a sensitivity of 1.0 and specificity of 0.88, and the calculated cut-off point for CTO was 6.23 nmol/min•kg. Similarly, ROC analysis indicated that FDA/PI (AUC = 0.49) and OCR/DNA (AUC = 0.58) were not predictive of post-IAT outcomes, while OCR/DNA/ISI (AUC = 0.72) was somewhat predictive (Fig 2). *IE dose* and *OCR dose* were highly predictive of post-IAT outcomes, with *OCR dose* having better predictive ability (AUC of 0.96 vs. 0.94 with *IE dose*). No significant difference could be found between the AUCs for *IE dose* and *OCR dose*. For the different multivariate models, none of the additional variables reached any measurable impact. Age seems to have a slight impact as there was a significant negative correlation between *IE dose*, *OCR dose* and age.

**Table 1:** Comparison of demographic, isolation, and quality assessment variables between insulin dependent and independent clinical transplant outcome 6–12 months post-transplant.

	Insulin status		
	Dependent	Independent	p-value
*N*	25	10	-
*% female*	76%	80%	-
*Age (years)*	39 ± 2	31 ± 5	0.139
*BMI (kg/m* ^*2*^ *)*	26 ± 1	21 ± 0.9	0.006^a^
*Trimmed pancreas weight (g)*	74 ± 7	72 ± 8	0.956
*IE/g pancreas*	3,579 ± 517	6,393 ± 897	0.046^a^
*Purity (%)*	27 ± 3%	44 ± 7%	0.019^a^
*Tissue volume (TV; mL)*	10 ± 1	13 ± 3	0.674
*Islet size index (ISI)*	0.86 ± 0.06	1.2 ± 0.1	0.009^a^
*FDA/PI (% viable)*	92 ± 1%	92 ± 2%	0.956
*OCR/DNA (nmol O*_*2*_ */min•mg DNA)*	110 ± 6	115 ± 5	0.476
*OCR/DNA/ISI (nmol O*_*2*_ */min•mg DNA)*	140 ± 10	104 ± 12	0.043^1^ ^a^
*IE dose (IE/kg)*	3,800 ± 250	7,500 ± 660	< 0.001^a^
*OCR dose (nmol O*_*2*_ */min•kg)*	4.3 ± 0.3	8.9 ± 0.9	< 0.001^a^

Data are means ± standard error or percentage.

^a^significant difference (p< 0.05) between groups as calculated by a Mann Whitney non-parametric test.

**Table 2:** Summary of cases where *OCR dose* correctly predicted clinical transplant outcome, whereas *IE dose* did not.

Case #	OCR/DNA (nmol O_2_/min•mg DNA)	OCR dose (nmol O_2_/min•kg)	IE dose (IE/kg)	Insulin status at 6–12 months
25	76	4.1	5,199	Dependent
33	96	5.1	5,314	Dependent
35	88	6.0	6,586	Dependent
32	97	6.2	6,147	Dependent
11	142	6.5	4,414	Independent

## Discussion

As islet transplantation moves closer to becoming standard-of-care, *in vitro* islet quality assessment assays that can be performed routinely and prospectively to predict CTOs are needed. In our study, we present data on the utility of an assay based on cellular OCR which may be helpful in predicting rates of II in clinical IAT. Both *IE dose* and *OCR dose* were highly predictive of CTO. The *IE dose* in our cohort of patients was more predictive of CTO than a past cohort presented by Anazawa *et al* [10]. Therefore, it is possible that in a larger cohort *OCR dose* would be significantly more predictive than *IE dose* especially in those patients who receive a marginal *IE dose* as illustrated in Table 2.

The concept of transplanting an adequate *IE dose* to achieve II is intuitive, and it has been proven to be important in clinical practice [1, 2, 4, 7–9, 25–28]. Based on experience and data with clinical intraportal transplantation, the minimum required dose to consistently achieve diabetes reversal has been found to be approximately 5,000 IE/kg for IAT [8, 9, 17, 29] and 10,000 IE/kg for islet allotransplant [1, 2, 4, 8, 25–28]. The effect of viability also has been recognized and islet membrane integrity (as measured by FDA/PI staining) is a standard islet product release criterion. There are two major limitations in the current approach: 1) the minimum *IE dose* is not adjusted for islet product viability, and 2) the currently-used viability assay (FDA/PI) is insensitive [5, 6, 13]. An islet viability value <70% is rarely reported, which may indicate to some that there is no need to make an adjustment for islet product viability when considering the *IE dose*. However, recent reports with more reliable islet viability assays, such as OCR/DNA [6, 13, 14], demonstrate that there is large variability in islet viability as measured by OCR/DNA but not as measured by FDA/PI, and suggest the need to account for viability in the *IE dose*.

We have previously used the mouse bioassay to study the utility of OCR and OCR/DNA to predict diabetes reversal with high-purity human and rat islet preparations [13, 15]. Published results demonstrate that when used together these parameters can predict diabetes reversal in mice with high specificity and sensitivity. When used in combination with *OCR dose*, OCR/DNA values of 150 nmol O_2_/min•mg DNA provided a reliable threshold above which diabetes reversal was predicted in rat islet transplants into immunosuppressed mice [16]. However, in this study, OCR/DNA alone was not predictive of CTOs (Fig 2D). A reason may be in the difference in the transplant site (renal subcapsule in mice vs. liver in humans). Preparations with low OCR/DNA indicate a larger fraction of dead or dying tissue. It may be that transplanting large quantities of dead or dying tissue into a confined space, such as under the renal capsule, may more greatly impact the engraftment of the viable tissue by sequestering an inflammatory response directly into the site of transplant and or by increasing the diffusional distance for oxygen. This same phenomenon may not occur following intraportal transplant since the tissue is not confined to a small space, but rather distributed throughout a large organ. In our study, *OCR dose* was highly predictive despite the wide range of product purity in the preparations examined (10–95%). Interestingly the data showed recipients that achieved II had a significantly higher purity than those who were dependent 6–12 months post-IAT (Table 1) suggesting that low purity may be associated with worse transplant outcomes.

Initial observations at the University of Minnesota suggest that a larger transplanted TV (typically due to lower purity) may itself be associated with worse CTOs. Our group has found that large TVs result in increased post-IAT portal pressures, which are directly associated with higher complication rates (portal venous thrombosis and bleeding). In a separate ROC analysis of a large cohort of TP-IAT patients (n = 233), our group has identified an increased risk of portal venous thrombosis at >26 cm H_2_O, which is more likely to be exceeded with transplanted TVs of >0.25 mL/kg [30]. Since data on TV is collected for all preparations, use of information on TV may enhance the predictive utility of *IE* and/or *OCR doses* and should be studied further.

Islet size has also been implicated as predictive, with smaller islets expected to result in better CTO, especially at a marginal *IE dose*. In a recent publication [19], patients who achieved II had a significantly smaller ISI (i.e. on average islets with diameter smaller than 150 micrometers). Islet size in combination with OCR/DNA (OCR/DNA/ISI) was also recently associated with better outcomes in a porcine islet to mouse xenograft model [22]. In this model, higher OCR/DNA/ISI was associated with a greater probability for diabetes reversal. In the cohort of patients reported in the present study, the results were contradictory as ISI was significantly higher and OCR/DNA/ISI was significantly lower in patients who achieved II and the OCR/DNA/ISI ratio was not as good at predicting CTO as *IE* or *OCR dose*. We do not have a clear explanation for this difference, but it is conceivable that the effect of islet size is marginalized when very high *IE doses* are transplanted. In other words, the total number of viable and functional β-cells transplanted (e.g. at the periphery of larger islets) exceeds the minimum number required for II, and more than accounts for the number of non-viable and/or non-functional β-cells within the core of the larger islets. However, in a marginal *IE dose* transplant, having smaller islets with a minimized number of hypoxic β-cells in their core may have a substantial impact on CTO [19, 20].

There are some limitations of this study. Calculation of the *OCR dose* is based on the *IE dose* and thus the values have some inherent uncertainty since they are based on operator-dependent manual counts. In addition, rather than correlating *OCR dose* only with categorical CTOs, it may also be appropriate to quantify the reduction in exogenous insulin requirements and also account for recipient factors, such as varying insulin sensitivity between individuals. These improvements will aid in the case of islet allotransplant, as we predict that this model will be more difficult to correlate CTO with characterization assays due to confounding factors such as the need for immunosuppression therapy, potential rejection, and the high possibility of multiple preparations being transplanted into a single recipient.

In conclusion, these data suggest that FDA/PI and OCR/DNA are not predictive of CTO following IAT, while OCR/DNA/ISI may be of some utility. When *IE dose* (which is predictive of CTO) is combined with the qualitative assay OCR/DNA (*OCR dose*), we are able to achieve a highly predictive measurement which may be useful for the prospective evaluation of the quality of islet preparations prior to clinical transplantation. Future studies will involve optimizing the predictive capability of *OCR dose* in the IAT model and examining the utility of these islet product characteristics, specifically *OCR dose*, on the more challenging case of islet allotransplant.
